# Immigrant-critical alternative media in online conversations

**DOI:** 10.1371/journal.pone.0294636

**Published:** 2023-11-30

**Authors:** Victoria Yantseva, Davide Vega, Matteo Magnani

**Affiliations:** InfoLab, Department of Information Technology, Uppsala University, Uppsala, Sweden; Caleb University, NIGERIA

## Abstract

In this work, we explore the role of immigrant-critical alternative media in shaping collective emotions and users’ evaluations of the immigration issue, using a conversational approach and an empirical case of Flashback, a prominent Swedish online platform where many immigration-related discussions take place. Our text and network-based analysis of more than 9,000 conversations during the last election period reveals that the platform users consume and distribute diverging types of media content across a wide ideological spectrum which, however, has a limited influence on the evolution of conversations and users’ stances in the immigration debate. Nevertheless, we find that the conversation networks with alternative media content tend to include more negative evaluations of the immigration issue, attracting fewer participants and lasting less than other conversations. We contextualise our findings using Collins’ Interaction Ritual Chains (IRC) theory and discuss the conditions under which such online conversations can produce high user involvement and, potentially, participants’ radicalisation.

## 1 Introduction

With the recent rapid rise of social media networks and online news media, social research has dedicated considerable effort to grasp the consequences of online communication and almost barrier-free distribution and consumption of media content, with the growth of right-wing platforms and news outlets as one particular outcome of these processes. While the consequences of audience exposure to alternative and right-wing media content are many, such as, for instance, the emergence of alarmed citizens [1], the effect of such content on users’ emotions and immigration attitudes is one of the riddles that social research has attempted to solve. For instance, the existing studies have focused on users’ exposure to alternative media and users’ reactions to such content in their news feeds, without paying much attention to neither already segregated or radicalised environments, or the cases where such content is consumed as part of the ongoing interaction with others.

This study explores the use of Flashback, a Swedish online forum with more than 1.5M registered users as of February 2023, which makes it one of the biggest online platforms in Sweden. Flashback is used by a large part of the Swedish population to discuss a wide range of topics, starting from gardening and PCs, and all the way to drug use, conspiracy theories or radical ideologies. While Flashback claims to ensure freedom of speech and users’ anonymity, it has also been used for personal attacks and other types of problematic online behaviours [2]. In particular, we focus on the “Immigration and integration” [Integration och invandring] subforum, where many immigration-related discussions occur. This subforum’s case is particularly interesting since previous research has demonstrated that biased and Islamophobic narratives are quite common on the platform ([3, 4]), which makes it a relevant object of study as an example of a fringe online community.

With the goal of investigating the role of immigrant-critical alternative media in shaping collective emotions, in this work, we measure the impact of such content on the dynamics of conversations on the subforum and the users’ evaluations of immigration issues. Working on a four-year snapshot of the data, we are interested in exploring if the dominant type of content shared on the platform influences, or is influenced by, the user stances or the structure of the conversations emerging.

The topic of the study is important for several reasons: because such online discussions have been shown to enable offline mobilization or support for protest participation [5, 6], because right-leaning echo chamber-like environments may provide a breeding ground for the spread of not only alternative media content, but also disinformation and conspiracy theories [7, 8], and, finally, because the consumption of content from right-wing alternative media has been shown to have a profound effect on immigration attitudes [9]. In many ways, this research complements the existing studies on the formation of collective emotions, diffusion of content from alternative media in online networks and the evolution of conversations online.

Using a dataset of 270k messages from the Flashback forum spanning the last election cycle in Sweden (2018–2022), we investigate if the dissemination of links leading to immigrant-critical alternative media can be one of the potential drivers for the conversations on this subforum. Then, we perform qualitative resource labelling to identify alternative media content shared on the subforum, and study the evolution of conversations with respect to their pace and emotional states of the conversations’ participants. We make the following contributions:

- Theoretically, we draw on the sociological literature on emotional group dynamics, and in particular, Randall Collins’ interaction ritual chains theory (IRC) [10]. The IRC theory provides a framework to understand users’ interactions on the forum, in this work denoted as conversations, and link sharing as specific types of social actions to achieve group solidarity and emotional synergy that potentially could lead to participants’ emotional (or affective) mobilization, or the production of “emotional energy” in theory’s terms [10] (See Section 3). In this paper, we apply this theory to mediated forms interactions and discuss the possibilities of translating this approach to online contexts.
- Methodologically, we explore the evolution of conversations on the subforum with the help of a computational approach and use a range of methods for text and conversation analysis, such as neural networks to classify users’ stances with respect to the topic, and social network analysis to reconstruct the conversations from message threads and analyze the properties of the resulting conversation networks. We describe how conversations are modelled in Section 4.1.
  Another methodological challenge is to evaluate quantitatively the direct outcomes of alternative news content sharing beyond general sentiments and network clustering. To that end, we use a custom stance classification model, which makes us able to identify how users’ stances on immigration differ depending on the types of content shared in the conversations (See Section 4.2).
- Further, we investigate the patterns of circulation of alternative media content *inside* an already segregated and fringe right-leaning environment (Flashback), which is especially relevant given the proliferation of such communities in the online space (See Section 5.1). This is important as the existing research has mostly provided evidence for the circulation of trusted versus problematic content and resulting user clustering in social networks in general, thereby paying less attention to the environments that may be more susceptible to such information, and where such information may have a stronger effect on users’ perceptions. Our analysis shows that, quite surprisingly, platform users engage with diverse types of media content, from mainstream national publishers to radical right online sources. While negative appraisals of the immigration topic are indeed over-represented in conversations with alternative media content, such conversations nevertheless tend to have lower user engagement.
- Finally, our ambition is to extend the existing evidence on the topic of alternative media effects by looking at the problem in conjunction with the conversation dynamics. One may suggest that the latter inevitably affects the way users perceive and react to the information provided as part of the conversation. In other words, users not only react to alternative news content shared but also take into account the previous content of the conversation, something that has mostly been overlooked by the existing research that has mainly focused on retweet networks or engagement with information from user feeds. In our analysis, we find a limited effect of alternative media content on the pace and tonality of conversations and thus view them rather as an element of a collective symbol system rather than an effective conversation driver and an instrument that can enable users’ radicalisation in the fringe online communities.

## 2 Related work

### 2.1 Alternative media content on social media platforms

The existing research demonstrated that platforms play quite a significant role in news sharing and distribution of alt-right content [11]. In the Swedish context, alternative media readership was found to stand out from a sample of other Northern and Central European countries [11], which makes it hard to underestimate the role of this information source and the potential effects it may have on the readers’ attitudes and political orientations. On top of this, alternative media readership was associated with distrust in the mainstream media, however, it also needs to be mentioned that it was found to supplement rather than completely replace traditional news outlets [12]. On social media and, in particular, on Twitter, alternative media presence was described in terms of “echo-system” where the same alternative media content was disseminated by an ideologically diverse set of actors [13]. On Facebook, links to Swedish alternative media were found to constitute almost one-third of all URLs shared during the 2018 election period, with link engagement levels almost on par with that of traditional media [14]. Even more so, the existing research demonstrated that Swedish right-wing actors were more successful in engaging the public during the 2018 election in comparison with their established counterparts, in particular, by means of resorting to offensive language and negative emotionality [15]. A similar study in the US setting also found that ideologically extreme news sources enjoyed the highest engagement levels on Facebook, despite that well-known mainstream publishers had larger audiences, since the latter did not necessarily imply high engagement with their news content [16].

When it comes to the use of alternative versus mainstream media in right-wing online environments, the existing research pointed out that, quite surprisingly, the use of and references to mainstream media sources are just as popular as those to alternative ones [17, 18], suggesting that right-wing audiences consume different types of content despite the dominating narrative of mistrust into the established media. Thus, the research suggests, despite the circulation of illiberal or radical opinions inside fringe and echo chamber-like environments, such platforms still allow for the dissemination of cross-cutting content and can nevertheless serve as deliberative spaces [19].

With regard to the dynamics and patterns of alt-right content sharing, a multi-platform comparison of alternative versus mainstream media news spread demonstrated that some of the fringe communities across the platforms generate a disproportionately high volume of alternative links sharing events that also seem to influence the emergence of the same content on other platforms [20]. Another study by Wang et al. that considered trustworthy and untrustworthy news sources instead of mainstream versus alternative ones arrived at a similar result and suggested that small communities can become extremely effective in pushing news stories to other communities [21]. Another relevant contribution to the existing evidence was made by Luna et al. who highlighted the differences in the dissemination of mainstream versus alternative media on Facebook: while alternative news content sharing was steadily increasing with time after the publication, mainstream news content sharing, in contrast, followed a burst and decay pattern [22]. Despite the existing evidence, little is known about the impact of alternative media content on the dynamics of discussions in fringe communities and whether such content reinforces or even produces more negative attitudes and narratives about the discussion topics. Some of the existing exceptions is a study by Introne et al. who provided a description of how pseudo-knowledge comes into being as a result of collaborative effort in the forum discussions [23].

### 2.2 Collective emotions and emotional contagion

Quite logically, negative and emotional content was shown to catch more users’ attention and disseminate more intensively [24, 25]. Likewise, the content diffused by alternative media was shown to be more negative and emotionally charged in comparison with the mainstream media content [26] and was shown to be more likely to express such emotions as anger and disgust [27, 28]. On Facebook, immigration- and security-related posts by right-wing actors were found to be especially likely to cause “anger” reactions and further sharing of those posts [29]. However, Berger and Milkman [30] demonstrated also that the relationship between emotion and virality is more complex than that: in particular, some emotions, such as sadness, might actually have a reverse, deactivating, influence on content virality. Rather, the emotions that evoke high arousal (no matter if they are positive or negative) are the main driving force for the diffusion of any type of content in online networks.

Some of the existing studies, mostly based on the results of experiments and agent-based modelling, also suggested that emotional content leads to higher arousal [31], while the valence of posts published on Facebook was found to depend on the valence of posts previously seen by a given user [32]. Another observational study of Twitter users and their followees’ posts arrived at a similar conclusion, thereby giving evidence to the presence of emotional contagion [33]. Some works suggested also that negative emotions, such as anger, expressed in the initial social media content, are more contagious than positive ones, such as joy [34, 35]. Yet another study demonstrated that, although the emotional tone of users’ messages is usually adapted to that of the chat, chat’s emotion tone can be characterized as persistent, rather than fluctuating towards particular negative or positive emotions [36]. Finally, concerning the connection between emotions and online deliberation, the previous research has distinguished constructive and non-constructive emotions, with the former being the only type of expression that can positively affect the quality of online deliberation [37]. A case study of the Chinese online platform Weibo, in contrast, has demonstrated a limited possibility for the platform to serve as a space for deliberation, with emotional content being a limiting factor [38].

What distinguishes our approach from the earlier effort is that we focus not only on the process of emotional contagion online in general (e.g. through news feeds and following/befriending other users) but rather on the group dynamics emerging as a result of users’ participation in online conversations that may lead to different dynamics and consequences of exposure to different types of emotions in news content.

### 2.3 Conversation dynamics online

With regard to the properties and dynamics of conversations, the existing research focused on several aspects such as the roles of individuals [39, 40], factors that make conversations interesting [41], conversations’ length and user participation prediction [42], or even the outcome of conversations in terms of demonstrated prosocial behaviours, such as social support, cohesion or mentoring [43]. Another piece of work employed a conversational approach to model content disputes on Wikipedia talk pages and developed a model for dispute escalation prediction task [44].

Since many of the existing works are based on the assumption that threads themselves represent distinct conversations, such a thread-based structure was found to enable higher reciprocity between users [45]. Further, work by Bagavathi et al. attempted to study conversations on Gab as an example of cascades and identified several types of cascades typical on the platform [46]. A similar approach to model conversation dynamics as a cascading process was proposed by [47]. Yet another approach to conversation reconstruction also took into account the linguistic features of the messages to determine the reply-to relationship between them [48]. The study most relevant to our empirical case, even though it does not explicitly focus on conversations or alternative news content, is the study by Caetano et al. [49] who explored structural, temporal and user engagement properties of “attention cascades” with falsehoods in non-political versus political WhatsApp groups. In particular, they found that the latter generate deeper and wider cascades [49].

Finally, it is also worth mentioning the effort to adapt Randall Collins’ micro-sociological approach to account for mediated forms of interaction and online communication, even though it was initially created with only offline interactions in mind. For instance, DiMaggio et al. applied Collins’ IRC theory to explore users’ behaviours online. This study also used a thread-based approach to online interactions between the users and explored the predictors of thread length, with the latter serving as a way to evaluate the interactions’ success [50]. Another interesting example is the work of van Harpenen et al. who applied methods of image recognition to develop a typology of mediated interaction rituals using a dataset of Instagram pictures related to the Black Lives Matter movement [51]. They proposed that such visual content served as a tool to connect with the movement despite geographical and physical separation, which also speaks in favour of the theory’s relevance for mediated types of communication.

## 3 Theoretical framework: Online conversations and collective emotions

Our work draws on Randall Collins’ micro-sociological approach and, in particular, Interactional Ritual Theory (IRC), a framework to study collective emotions that come into life as a result of social interactions [52]. In his take on conversations, Collins largely follows the tradition outlined by Goffman and the Conversation analysis (CA) theory [53]. The latter views conversations as a type of social action organised in a specific way. In particular, it presumes turn-taking and sequential ordering of interactions—participants talk in turns and switch the roles of speakers and listeners [54]. Moreover, it implies the interdependence between the participants and contextual character of their interaction—participants take into account what others say to be able to respond, and, by responding, they renew the conversation context [55]. In the context of online conversations, distinct utterances of the conversation participants are represented by the messages published as part of the same conversations that also appear on the communication platform sequentially. Just as in off-line conversations, participants engage in turn-taking—they publish utterances and also read the utterances of others, and by doing so, engage in understanding and shaping the context of the conversation.

In accordance with the IRC approach, conversations can also be represented as one type of social action that can produce emotional synergy and group solidarity [56, p. 197] in cases when the necessary conditions are fulfilled, in particular, group assembly and barriers to outsiders, as well as common mood and focus of attention (for more details on the key principles of the IRC theory, see e.g. [57]). Collins’ take on conversations is in many ways coherent with our own understanding of conversations as an evolving chain process where an interaction depends on the previous interactions in the chain, which can be perfectly summarized by the following excerpt:

“At any particular moment, people are speaking certain words or thinking certain thoughts; the thoughts that go through one’s head are internalized from previous talk with other people; more innovative thoughts are assembled out of the ingredients of verbal ideas already internalized. The world is a network of conversations, and what people think at any point in it is a product of what has circulated in previous conversations. There is a crucial emotional component: ideas are better remembered, and make more sense, if they were associated with emotion when they were previously talked about” [52, pp. 303–304]

Contrary to the Habermasian statement about the deliberative and rational character of information exchange [58, p. 415], one can suggest that such conversations do not necessarily need to follow any rational or instrumental value—in other words, these discussions can be described as “[…] emotional, symbolic or value-oriented behaviour” [10, p. 205], which distinguishes rituals, including the mediated ones, from purely instrumental types of actions. In particular, users’ main motivation for the participation in conversations can lie in the production of particular emotions and “emotional solidarity with the group” [10, p. 215], rather than in reaching a consensus on a controversial topic or exchanging rational arguments for or against the discussed issue.

Following the same line of reasoning, an act of sharing content with other conversation participants can be described as both a symbolic and instrumental action. On the one hand, content sharing can be seen as a practice to initiate higher user involvement in conversation and thus higher mobilization and solidarity, and, possibly, to invoke specific types of emotional reactions in those who consume such content. Content-sharing can also be used to justify particular attitudes or support particular opinions expressed by the users. Indeed, it has been shown that covert racist or biased attitudes are often justified with the help of argumentative and rational reasoning [59, p. 35]. On the other hand, content sharing can also serve as a specific collective and ritual symbol [10, p. 212], on par with, for example, specific jargon and language to talk about the immigrants [60]. The use of such symbols may be directed towards achieving higher group solidarity and the sense of group belonging enabled by the platform architecture.

In relation to the right-wing, populist or reactive movements specifically, Collins suggests that those are more prone to emotional mobilisation: “[…] reactive movements […] are easier to mobilize, and generally more emotionally aroused than positive movements seeking a better future […]” [61, p. 305]. This, Kemper adds, is even more relevant in cases when there exists an out-group (in this case, those labelled as “immigrants”), so that the focus of common attention, which is a necessary trait of any ritual, can be directed at those considered enemies [56, p. 177].

The question of whether Collins’ sociology of emotions is applicable to online contexts has lately been subject to debate. Collins himself has dismissed the idea that online environments can generate emotional solidarity due to the participants’ temporal and spatial misalignment [62]. Yet, some of the recent studies have successfully demonstrated the theory’s applicability to online contexts and found evidence for the emergence of collective emotions and group solidarity. A particularly interesting example is a study by DiMaggio et al. who analysed forum discussion threads and provided evidence that some of the theory’s propositions hold even for online contexts and may be illustrative of some traits of human communication in general [50].

Nevertheless, mediated conversations such as those on Flashback differ from offline and in-person conversations in a few ways. The most obvious one is, of course, the above-mentioned temporal and spatial misalignment. The conversation participants are not only physically disconnected from each other, but they also participate in conversations asynchronously, with intervals of several seconds or even days. Despite such a misalignment, the ordering of interactions and reply-to functionality allow users to infer the previous content of conversations without losing too much context. On top of this, despite temporal misalignment, one can nevertheless suggest that online conversations have their own pace, which is an important component in generating emotional synergy in Collins’ approach [63]. As it follows from our results (See Section 5), the vast majority of utterance exchanges in the subforum we studied happen within a quite narrow time span of minutes. One may suggest that, if the pause between messages is too large, then the conversation pace gets interrupted—that is why our approach, in contrast to the earlier works that equate threads to conversations, disentangles various conversations that take place as part of the same thread. For instance, if the thread topic catches the users’ attention, they might not only participate in the conversation by posting an utterance, but also stay online and follow up on how the conversation unfolds, which creates the conversation entrainment mentioned by Collins, and, potentially, generates emotional energy.

Furthermore, the format of online conversations is underpinned by the platform design or, in other words, the logic and functionality of the platform. If offline rituals can take numerous forms, online interaction rituals, in their basic form, presume the written form and permanency of the messages (if those, of course, are not based on disappearing or voice messages). Another aspect related to the platforms’ design is their functionality—for instance, Facebook allows liking others’ messages, while Flashback does not—in other words, platforms constrain users’ behaviours or, rather, allow users to act only in specific ways, which shapes the ways online conversations may develop. One such platform affordance that is quite common to almost all platforms is exactly cross-platform content sharing—or the possibility for the users to circulate and access additional digital content, such as external websites, photos and videos, which can provide additional context or support the speaker’s utterance.

It also needs to be mentioned that online conversations are also different from other types of online communication, such as for instance, retweeting others’ messages or commenting on other users’ posts or content. In the case of retweets, the retweeting user does not add any substantive content on top of the content already provided by the retweeted message. In the case of user comments, then, the comment author does not need to take the previous comments into account—in other words, they can post the comment irrespective of other users’ expressions. On the contrary, some other forms of online communication, such as, for example, instant messaging services such as WhatsApp or e-mail, can also be described as online conversations, since they presume that the replying user needs to be aware of the previous content for the conversation to make sense. On the other hand, not every online conversation can be described as an online ritual, since it might not necessarily meet the requirements of common mood and sense of group belonging [61].

Finally, one can suggest that what Collins himself calls the production of “emotional energy” as an outcome of any ritual can be operationalized in at least two different ways using the idea of *entrainment*, or users’ engagement with and involvement in the ritual [64]. The conversation entrainment can be expressed, for instance, in terms of conversation length—a successful conversation may be longer, or in terms of its pace, or rhythmic coordination [63]—the shorter and more consistent the interval between the posts, the more engaged with the conversation the users are. The second way is through measuring emotional mobilisation, or the co-evolution and intensification of shared emotions generated in the conversation [64].

## 4 Data and methods

Our empirical case for the analysis is represented by the Flashback forum that was briefly described in the Introduction. Flashback as an online platform has a typical forum structure: it is nested into subforums that discuss specific topics, such as e.g. “Politics”, “Cars” or “Family and children”, which the subforums consist of a number of threads, that, in their turn, are populated with users’ messages. Similarly to many online forums, users can quote other users’ messages and post links to external sources. However, in contrast to other social media platforms, the forum users cannot “like” or repost other users’ messages, while the thread structure does not imply message nesting, such as, for example, on Facebook: all of them are posted one after one, and it is thus impossible to comment on other user’s comment, except for using the quote function.

The data from the Flashback forum were collected in December 2022 using the R package *httr* [65] (replication code for data collection and analysis is available at https://github.com/victoria-yantseva/conversation-networks/). In particular, we scraped all discussion threads in the subforum “Integration and immigration” [Integration och invandring] that were posted during the last election cycle starting from 18th of January 2019, when the new Prime minister was chosen, until the 12th of August, 2022, which was the last day for the parties to register their participation in the elections. The choice of this time frame was dictated by the assumption that elections not only change users’ interaction patterns but also the ways they talk about immigration, which is a pressing and highly debatable topic in the Swedish context.

Collected data included the following information: thread titles, usernames of the message authors and quoted users (similar to the reply function on Twitter), as well as message IDs, texts and dates of publications. In total 270k messages from 4,692 discussion threads were collected. Table 1 summarizes the main properties of the collected dataset. All analyses as part of this study re-used data already publicly available on the Web and published anonymously. However, to preserve users’ integrity, the analysis was performed on the group (aggregated) level, and we avoided pointing out individual users in the manuscript text.

**Table 1:** Dataset summary.

Number of posts	276,716
Number of threads	4,692
Avg. number of posts per thread	59
Number of unique users	14,366
Avg. number of posts per user	59

The need for an ethics review of this study was waived by the Swedish ethics review authority (Etikprövningsmyndigheten) which concluded that ethics permission for this study was not needed (Decision No 2022–05390-01 by the Regional ethics review authority in Linköping). Users’ consent to data collection and processing was not obtained, since the Flashback forum guarantees users’ anonymity, and it is thus impossible to identify and contact individual users.

Our subsequent pipeline for data processing and analysis included the following steps:

- Extraction of edge lists and reconstruction of conversation networks;
- Extraction and coding of URLs;
- Stance classification and sentiment analysis;
- Statistical and network analysis.

### 4.1 Conversation reconstruction

Our two primary assumptions for the extraction of conversations is that, on the core level, a) a discussion thread consists of one or several conversations, depending on the frequency of interactions, and b) that such conversation can be represented either as a temporal network consisting of interconnected messages or, on an aggregated level, of users and links between them. However, in contrast to offline conversations that share the same spatial and temporal dimensions, online interactions are asynchronous. As a consequence, not all forum users participate in the conversations simultaneously. We note that more than two-thirds of all messages (third quartile) are published within a rolling window of 27 minutes (Δ*t* < 27), and we create a link between any pair of messages in a thread that is published within this time span, also known as Δ*t*-temporally adjacent messages [66]. We validate the robustness of this rolling window choice by testing alternatives: the thresholds that are two times shorter (13 minutes) and two times longer (54 minutes) to account for the fact that some of our results may depend solely on the choice of the rolling window. Further, we replicate the whole analysis for the conversations created using the three rolling windows and confirm that our results hold irrespective of the chosen threshold (unless specifically mentioned in Section 5).

To provide an example, if user A posts a message at time *t*_1_, and user B at time *t*_2_ (within 27 minutes after A’ message), then we assume that user B has read user A’s message and potentially taken its content into account while writing their own message. Thus, we create a link from B’s to A’s message. Additionally, we create also a link from B’s message to any other message quoted in it (similar to a reply on Twitter), irrespective of the amount of time that has passed, since this indicates a direct reference to the message previously published.

Thus, we may assume that a conversation, on an aggregated level, tends to capture the amount of information from a given thread that a given user will consume. Moreover, our approach to conversations takes into account the sequencing of messages, which helps to account for the co-evolution of users’ narratives. Lastly, since we are also interested in studying the properties of conversation networks, we explore a range of the resulting networks’ basic characteristics. Those include: average clustering coefficient, total number of edges and nodes, and edge density, all available as part of the NetworkX package in Python [67].

### 4.2 Classification of stances and sentiments

To quantify the sentiments on and evaluations of a specific topic (immigration and corresponding policies in Sweden), we use a notion of *stance* and follow the definition of stance-taking as a subjective evaluation or appraisal of a specific target [68, p. 142, 153], “explicitly mentioned or implied within the text” [69]. Although this task is closely related to that of classic sentiment polarity detection, the difference between the two is that sentiment analysis is concerned with identifying the sentiment polarity of a text in general, whereas stance classification requires a specific target (be it an entity, topic, claim etc.). For instance, the following statement “It is frustrating that we still need to fight against implicit ethnic discrimination of newcomers in our country” can be described as having negative sentiment polarity, but at the same time taking a positive or supportive stance with regard to the immigration topic.

Thus, in this work, we aim at identifying user *stances* with regard to the immigration issue. We use a two-step machine learning approach trained specifically on the data from the Flashback forum. The details on model training and selection are discussed in detail in [70]. In short, on the first step, our approach identifies on-topic and off-topic messages, since, as mentioned above, we are only interested in messages discussing immigration. In the second step, particular stances are determined (two classes: negative and non-negative, the latter includes neutral and positive messages). Choosing a two-class approach allows both to compensate for class imbalance (positive messages are under-represented in the corpus, accounting for only 5% of the training dataset), as well as distinguish negative rather than moderate expressions about immigration, whether positive or neutral.

Stance classification is further supplemented with an unsupervised sentiment analysis using the VADER dictionary [71]. Since this is a dictionary-based and unsupervised approach, it suffers from the limitations typical for this family of sentiment analysis methods such as lexicon narrowness and domain independence. However, our previously performed experiments demonstrated its reasonable performance for social media texts in Swedish, with an accuracy of 62%, which is on par with the previously performed tests (e.g. see [72]).

### 4.3 Identifying immigrant-critical alternative media

We use the definition *immigrant-critical alternative media* and a relational approach suggested by Kristoffer Holt who states that “Alternative news media represent a proclaimed or (self-) perceived corrective, opposing the overall tendency of public discourse emanating from what is perceived as the dominant mainstream media in a given system.” [73]. Other important traits include alternative news producers, in particular, non-professional actors, such as readers and activists; alternative news content, or the narratives that are perceived as counter-hegemonic and marginalised in the mainstream discourse; and, finally, alternative news organisations, in particular, in low-cost formats, such as blogs and webpages [73]. In contrast, we define *mainstream media* as established news organisations that rely on the work of professional journalists, adhere to ethical and professional journalistic standards and have an editor or an editorial board responsible for the content produced.

Our resource labelling process begins with the extraction of the most used domains mentioned by the users. In total, we extract 332 unique resources that comprise only 1.5% of all unique resources shared on the subforum in 2019–2022. Nevertheless, those resources account for 75% of all links shared on the forum. Further, we exclude the resources that do not belong to the media category (for instance, websites of private companies, non-profit organisations and government agencies). The rest of the resources are evaluated manually based on a range of criteria following the approach described in [73], in particular a) non-oppositional or corrective stance in relation to what is perceived as mainstream immigration discourse, b) availability in high-cost formats (radio, printed press, TV), c) adherence to the press ethical norms and standards, d) a presence of editor-in-chief or editorial board, or similar actors responsible for the published content, e) belonging to a larger organisation or publisher.

Further, the resources are assigned one of the three labels: *mainstream/legacy media*, *immigrant-critical alternative media* (or simply *alternative media*) and *other media*. The *mainstream media* label is assigned to the resources that are evaluated positively on the criterion *a)* (as providing non-oppositional framing of the public agenda), plus on at least three other criteria. The procedure for labelling resources as *immigrant-critical alternative media* is exactly the opposite: those are evaluated negatively on the criterion *a)* plus on at least three other criteria. The rest of the resources are labelled as *other media*, since the distinction between mainstream and alternative media is rather blurred and represents a continuum, as noted by the previous research [73], rather than a set of distinct media format categories. The details of resource evaluation criteria and labelling are presented in the S1 and S2 Files. Finally, it needs to be mentioned that social media groups on popular platforms (e.g. Twitter or Facebook) are excluded from our classification since they account only for a minor share of links disseminated on the forum.

### 4.4 The dynamics of collective stances and emotions and the role alternative media

As a first step, we compare the characteristics of a) conversations with alternative media versus mainstream media links shared, and b) conversations with any type of links versus no links shared. To that end, we use Mann-Whitney U test that fits well to compare samples where data is non-normally distributed. The conversations are evaluated on a range of criteria, including:

- duration in hours;
- the ratio of messages with negative and non-negative stances;
- average stance probability;
- average clustering coefficient;
- the number of nodes and edges;
- edge density coefficient.

Further, we explore conversation entrainment by analyzing the conversation pace and the evolution of users’ sentiments. In other words, a successful ritual, as defined by the theory, can be measured in two different ways: first, by conversation pace, measured as the frequency of interactions in the conversation, and, second, by the evolution of sentiments and stances, which in the case of fringe or right-wing environments, can be denoted by the dominating negative moods and evaluations of the immigration topic. Even more so, a successful conversation may also imply the alignment of users’ pace of interactions and subjective expressions, so that each new message arriving as part of the conversation will be dependent on the previous message(-s) posted as part of the same interaction chain. To measure such conversation entrainment, we use two sets of mixed effects models with conversations representing a random (grouping) effect, aiming to detect whether there is a relation between the subsequent messages in each conversation. Our sample includes a total of 6,674 conversations, all of which have at least one observation after obtaining time-lagged data. All analyses are performed using lme4 package in R [74].

#### 4.4.1 Conversation pace

In the conversation pace model, we calculate the time intervals between each pair of adjacent messages (*Δt*). For example, if message A is published at 15:30 (*t*_1_), message B at 15:40 (*t*_2_), and message C at 15:50 (*t*_3_), then we consider that message C was published within the interval of 10 minutes from message B (*t*_3_ − *t*_2_ = 10), which we denote as Δ*t*_3_. Since we are also interested in checking whether users adapt their interaction pace to that of the conversation in general (or, in other words, if there is a temporal alignment in users’ interactions), we also take into account the interval at which the previous message was posted (that is, a lagged time interval Δ*t*_2_ = *t*_2_ − *t*_1_), which in our example is also ten minutes for message B. It needs to be noted that one of our predictor essentially includes a lagged outcome variable (which is known as multilevel autoregressive model), however, we avoid performing cluster mean centering since we are interested only in relationship between the predictor and outcome variables on average, or across our conversations (for a detailed discussion, see [75]).

We build three different models in order to compare their performance. The first version (see Eq 1) is a null model that includes only conversations as a random (grouping) effect and assumes no relation between the messages’ posting time intervals:

$$\Delta t_{ij}=\left(\alpha +u_{j}\right)+e_{ij}$$ (1)

where

*j* is an enumerator for our grouping variable (conversation ID),

*i* is an enumerator for the observations inside the group,

Δ*t*_(*i*−1)*j*_ is a response variable—predicted time interval for the *i*th observation in the *j*th group,

*α* is a global model intercept coefficient,

*u*_*j*_ is an intercept error for the *j*th group (conversation),

and *e*_*ij*_ is an error for the *i*th observation in the *j*th group.

The second model (Eq 2) adds the effect of the lagged posting time interval only. Thus, we include the message’s posting interval Δ*t* as a dependent variable and the previous message’s posting interval at Δ*t*_*i*−1_ as an independent variable. As mentioned above, our hypothesis is that users adapt their behaviour to that of the conversation, and, if interactions happen at a fast pace, then users will also be trying to adapt to the pace of preceding interactions.

$$\Delta t_{ij}=\left(\alpha +u_{j}\right)+\beta \Delta t_{(i-1)j}+e_{ij}$$ (2)

where

Δ*t*_(*i*−1)*j*_ is a posting interval Δ*t* for the preceding message *i* − 1 in the *j*th conversation,

and *β* is a global model coefficient for our independent variable (the posting interval for the preceding message).

Finally, in the third model, we add link sharing (any links, mainstream media links or alternative media links) at *t* − 1 as an independent (dummy) variable (See Eq 3). Specifically, we test whether links shared in the previous interactions have an impact on users’ interaction pace. For example, one could hypothesize that users may need more time to check the link content, and thus the conversation pace will be slower, or, on the opposite, that the distributed content would make users more engaged in the conversation:

$$\Delta t_{ij}=\left(\alpha +u_{j}\right)+\beta \Delta t_{(i-1)j}+\gamma l_{(i-1)j}+e_{ij}$$ (3)

where

*γ* is a global model coefficient for our independent variable (links shared in the preceding message),

and *l*_(*i*−1)*j*_ is an independent (boolean) variable for the links shared in the preceding message *i* − 1 in the *j*th conversation.

#### 4.4.2 User sentiments

In our sentiment model, we use the results of VADER sentiment analysis to explore whether users’ sentiments are impacted by the sentiments of posts previously shared in the conversations. For example, if message A has a VADER score of $V_{1}=-0.5$, and message B a score of $V_{2}=0.5$, then we want to evaluate if the score of message B (e.g., $V_{2}$) is dependent of the score of message A ($V_{1}$). This is due to the fact that we are interested in checking users’ emotional alignment, or, in other words, whether users adapt their message sentiments to that of the conversation in general, and preceding messages specifically.

Just as in the previous case, we test three model versions. The null model (Eq 4) includes only a random effect of conversations and assumes no relation between the preceding and subsequent message sentiments:

$$V_{ij}=\left(\alpha +u_{j}\right)+e_{ij}$$ (4)

where

*j* is an enumerator for our grouping variable (conversation ID),

*i* is an enumerator for the observations inside the group,

$V_{ij}$ is a response variable—predicted sentiment for the *i*th message in the *j*th conversation,

*α* is a global model intercept coefficient,

*u*_*j*_ is an intercept error for the *j*th group (conversation),

and *e*_*ij*_ is an error for the *i*th observation in the *j*th group.

The second model (Eq 5) adds the effect of the lagged message sentiments. In particular, we include the message’s sentiment $V_{i}$ as a dependent variable and the preceding message’s sentiment $V_{i-1}$ as an independent variable. This model allows evaluating whether there is an impact of preceding message sentiments on the subsequent ones, or, in other words, we test whether there is a co-evolution of user sentiments in the conversations.

$$V_{ij}=\left(\alpha +u_{j}\right)+\beta V_{(i-1)j}+e_{ij}$$ (5)

where

$V_{(i-1)j}$ is a sentiment score of the message *i* − 1 in the *j*th conversation,

and *β* is a global model coefficient for our independent variable (the sentiment of the preceding message).

Finally, in the third and last model (Eq 6), we add again a fixed effect of the media links shared in the preceding messages (any links, mainstream media links or alternative media links) at *t* − 1. In this model, we test whether media links shared in the preceding steps of conversation have an impact on the evolution of user sentiments.

$$V_{ij}=\left(\alpha +u_{j}\right)+\beta V_{(i-1)j}+\gamma l_{(i-1)j}+e_{ij}$$ (6)

where

*γ* is a global model coefficient for our independent variable (links shared in the preceding message),

and *l*_(*i*−1)*j*_ is an independent (boolean) variable for the links shared in the preceding message *i* − 1 in the *j*th conversation.

## 5 Results

### 5.1 Alternative media in the forum conversations

**Fig 1:**
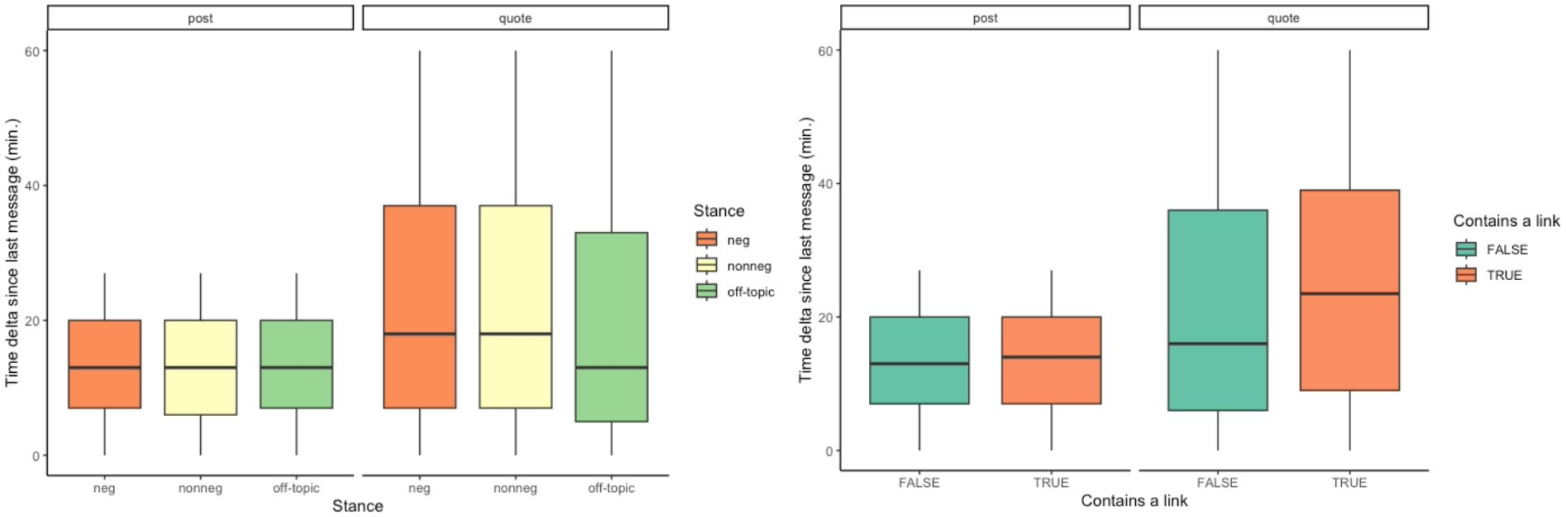
Conversations’ pace depending on edge type, links shared and message stances. (left): Interaction time intervals for different types of edges and message stances. (right): Interaction time intervals for different types of edges and links shared.

In total, we collected data from 4,692 different threads on the subforum that were started during the 2019–2022 election period. Those resulted in 9,304 conversations (with a rolling window of 27 min.), with an average conversation length of 28.4 and a median length of only 5 messages respectively, suggesting that the vast majority of conversations are rather short. As explained in the Methods section, our conversation networks include two types of edges, those between the messages posted in the time span of 27 minutes, and also those that denote replies to (or quotes of) other users, since the latter represent direct interaction between the conversation participants that can happen outside of the rolling window limit. Indeed, if we compare different types of edges, we find that user quotes take longer to be posted, and this is even more evident if we consider quotes that include any type of links (See Fig 1). Negative and non-negative messages take an equally long time to publish, while off-topic messages appear slightly faster in the conversations.

Our results indicate that, quite unexpectedly, links to alternative media (hereafter AM) comprise only a minor part of the links shared on the forum: only 12% of all conversations on the forum has at least one AM link shared, and more than every third one (34%) at least one mainstream media (hereafter MM) link shared. Likewise, of more than 14,000 unique users in the dataset, only a small part (8% or 1,147 participants) is responsible for the dissemination of alternative media content.

Speaking of particular sources shared, we note that mainstream media are circulated far more actively than alternative ones, which is somewhat striking since one would expect the forum’s audience to favour the sources that would problematize the existing immigration discourses (see Table 2). Nevertheless, we find that the shared resources cover the whole ideological spectrum with regard to the immigration agenda: from the more “conventional” narratives articulated by the largest national publishers (such as *SVT* and *Aftonbladet*) and all the way to the extreme right resources that at times resort to hateful and extremist rhetoric when talking about immigration and immigrants in Sweden (for instance, *Nordfront*). In between these two extremes of the spectrum can also be found resources that were assigned to *other* category, such as, for example *Nyheter Idag*, since they do not fully comply with our understanding of legacy media, but which at times are successful at mimicking the behaviour and publishing strategies of the legacy outlets. Somewhere in the middle of this spectrum can also be found international resources such as *Sputnik News* and *Russia Today* that clearly stand out in their interpretation of political and policy issues.

**Table 2:** Most frequently shared media sources.

Alternative media	Number of shares	Mainstream media	Number of shares
samnytt.se	1,131	svt.se	2,916
friatider.se	1,095	expressen.se	2,379
uvell.se	115	aftonbladet.se	1,993
petterssonsblogg.se	98	sverigesradio.se	1,253
detgodasamhallet.se	88	svd.se	888
nordfront.se	88	dn.se	870
snaphanen.dk	77	gp.se	611
swebbtv.se	44	sydsvenskan.se	310
thereligionofpeace.com	38	svtplay.se	262
breitbart.com	37	hemhyra.se	198

**Note**: The details of source coding and complete coding results can be found in Appendix 1 in S1 File.

Our hypothesis is that conversations, where alternative media content is shared, exhibit higher user activity since such content can spark and provide some ground for users’ discussions. Besides, it may reinforce and, potentially, intensify users’ already existing negative preconceptions about immigration and immigrants. We use the Mann-Whitney U test to check this hypothesis and compare the means of conversation samples where alternative versus mainstream media content is shared. Since different conversations have varying ratios of links shared, we perform a range of tests using different link ratio thresholds calculated as the number of links of a given type divided by the number of messages in the conversation (0.2, 0.4 and 0.6).

We find substantial differences in user stances on the immigration topic after comparing the characteristics that differentiate conversations with alternative links from those with mainstream ones (Table 3). In particular, conversations, where alternative media content is shared, tend to have a higher ratio of negative messages. At the same time, quite a counter-intuitive observation is that conversations with alternative media content also tend to be shorter, with a lower number of nodes and edges between them, but, at the same time, higher edge densities (*p* < 0.05 in both cases irrespective of the chosen threshold for the link ratios).

**Table 3:** Conversation properties conditional on the link types shared.

Conversation property	Conversations with AM links (vs. MM links)	Conversations with links (vs. no links)
Duration (hours)	Shorter	Longer
Ratio of messages with negative stances	Higher	Lower
Ratio of messages with non-negative stances	Lower	Higher
Average negative stance probability	No difference	Higher
Average non-negative stance probability	Lower	Lower
Average clustering	No difference	No difference
Number of nodes	Lower	Higher
Number of edges	Lower	Higher
Density	Higher	Lower

**Note**: The results of Mann-Whitney U test where we compared a) conversations with AM versus MM links, and b) conversations with any type of links versus no links.

We also decide to test an alternative hypothesis—for instance, one can suggest that link sharing in general, irrespective of link type, can affect the properties of conversations. Indeed, following the same procedure as the one described above, we find that link sharing in general makes the conversations longer, and leads to a higher edge density and a higher number of nodes and edges involved (Mann-Whitney U test, *p* < 0.05 irrespective of the chosen threshold for the link ratio in the conversations). Thus, one may suggest that content sharing can serve as a factor to spark conversations and users’ deliberations, irrespective of the type of the cited source.

Our last observation is also that conversations with any link types tend to have a lower share of negative evaluations of the immigration agenda in comparison with the conversations where no media content is shared (*p* < 0.05 irrespective of the threshold for the link ratios). Such conversations also tend to have a higher share of neutral and positive messages, which, however, holds only for conversations with a sufficiently high share of links in the conversation (*p* < 0.05 for hyperlink ratio over 0.4). Finally, we find that there are not only differences in the users’ stance polarities but also in their strengths denoted by the probabilities of belonging to a given category (negative or non-negative) calculated by the stance classification model. In particular, in the conversations with any type of links shared, stance probabilities are lower for the negative messages and higher for the non-negative ones (*p* < 0.05 irrespective of the threshold for the link ratios).

### 5.2 Conversation evolution and the role of alternative media content

Using mixed-effects models as explained in Section 4.4.1, we evaluate whether the previous conversation states at (*t* − 1) can be used to predict the subsequent conversation states at (*t*), which denotes conversation entrainment and users’ alignment with regard to the conversation pace and shared sentiments. On top of this, we evaluate whether link sharing (either alternative media, mainstream media or any links in general) has any impact on the evolution of conversations and the emotional states of its participants. In total, 6,674 conversation chains were included in the analysis. The minimal number of observations per conversation was 2 due to the need to include lagged observations in the models.

Most importantly, our results indicate that conversation entrainment is more visible if we consider the pace of interactions rather than message sentiments: in particular, the sentiments of the previously shared messages serve as significant (*p* < 0.05) but weak predictors for the subsequent message’s sentiments, suggesting a weak dependence between the sentiments of messages in the same conversation. The model that includes lagged message sentiments as a dependent variable performs only marginally better than the null model (see Table 4). This suggests that users’ emotional alignment is less discernible in comparison with conversations’ temporal alignment—conversation participants are not prone to picking up the emotional states of other participants. Note that, in this case, we use only the results of VADER sentiment analysis, which is a general sentiment analysis tool not capable of capturing the differences in users’ moods targeting the immigration topic specifically.

**Table 4:** The results of mixed-effects modelling.

Model	AIC	BIC	Marginal *R*^2^	Conditional *R*^2^
Pace model, null	104496.8	104528.1	0	0.17
Pace model, time interval lag 1	92193.8	92235.6	0.052	0.135
Pace interval, time interval lag 1, media links (any)	92189.3	92241.5	0.052	0.135
Sentiment model, null	414646.5	414677.8	0	0.036
Sentiment model, sentiment score lag 1	414569.6	414611.4	0.0003	0.034
Sentiment model, sentiment score lag 1, media links (any)	414569.8	414622.0	0.0003	0.034

In contrast, our model for the conversation pace is more successful in capturing the temporal relationship between the messages, and we find that the pace of preceding interactions can be used to predict the pace of subsequent interactions. Our model with lagged message posting intervals as an independent variable performs significantly better than the null model (ANOVA test, *χ*^2^ = 12, 294, *p* < 0.05, AIC 92,216 versus AIC 10,4507 respectively). In particular, lagged posting time intervals can serve as a significant predictor for the posting intervals of the subsequent messages (Estimate = 0.221, SE = 0.002, *p* < 0.05). We also note the positive estimate for the predictor, suggesting that conversations’ paces get longer with time, which also denotes a decaying pattern of the interaction pace. Nevertheless, our results indicate that we can infer the pace at which participants will be interacting by knowing how fast or slow the were communicating on the preceding steps.

Turning back to the question of the role of alternative media content in the conversations, we notice, once again, that link sharing, whether from alternative media or any other type of resources, does not have any sufficient effect on the conversation dynamics neither with regard to the conversation pace nor the dominating sentiments (*p* > 0.05 in both models). In our data, link sharing, irrespective of link type, has a significant impact on the conversation dynamics only when used as a predictor for conversation pace (*p* < 0.05), however, it does not improve model performance (AIC 92,193.8, conditional *R*^2^ 0.135 for the model with time-lagged post intervals data only versus AIC 92,189.3, conditional *R*^2^ 0.135 for the model also accounting for previously shared links).

## 6 Discussion

Summing up the results above, our main takeaway from the analysis is that Flashback’s audience circulates and consumes different kinds of digital content that covers the whole ideological spectrum with respect to the dominating immigration discourses, from large national mainstream publishers to radical far-right resources openly disseminating biased and racist narratives. This observation is consistent with the earlier results that provided evidence that even far-right social media group users consume diverging kinds of content [17]. Nevertheless, it is noteworthy that legacy media content is disseminated in an environment where mainstream narratives are considered invalid, as one would expect the platform’s audience to favour the resources that provide what is considered as the alternative and counter-hegemonic view of the immigration topic. Nevertheless, our conclusions confirm the earlier results that users even in similar fringe environments consume cross-cutting content, while the platforms themselves can nevertheless serve as spaces for deliberative talk [19].

Further, the use of mainstream resources in this setting can be explained, for instance, by the mechanisms of selective exposure and confirmation bias, whereby users might want to select and strategically use the content that corresponds to their pre-existing views (demonstrated by e.g. [76]), irrespective of where it was published. Yet another explanation can be that such mainstream narratives are simply denied and are used to exemplify a presumably biased representation of the immigration agenda by the mainstream media. However, since we lack the direct evidence to support this argument, one possible way to extend the existing analysis on mainstream media use in such fringe environments is to analyze what kinds of argumentation and reasoning (for instance, approval or contradiction) are used by the forum users to comment on the content originating from the mainstream media.

Nevertheless, despite we do not notice any users’ preferences for specific types of media content, in accordance with our expectations, we find that conversations where alternative media content is disseminated differ from those with the content from the legacy media in that they tend to have a higher share of messages with negative evaluations of the immigration agenda. However, this does not necessarily imply that such content *causes* more negative expressions. On the contrary, one may suggest that alternative media sharing may correlate with users’ preconceptions: in other words, those who have negative pre-existing attitudes to the immigration policies in Sweden may be more likely to circulate alternative media content in their messages, but this does not necessarily mean that those attitudes are adapted by other participants. Another counter-intuitive observation is that alternative media content has in a way a de-activating function with respect to the users’ engagement on the platform: although users’ expressions in such conversations tend to be more negative, the conversations with alt-right content tend to wane quicker and be shorter. This may partly be because they might help to quickly reach a consensus, or because they are distributed by the users who have peripheral roles on the subforum since we also find that only a small minority of users is responsible for the dissemination of the links leading to alternative media content. Thus, a possible avenue for future research is to explore user centrality and influence with regard to the consumption and dissemination of specific types of content.

### 6.1 Limitations of the work

One of the constraints with regard to the chosen methodological approach is of course that only a subset of the most frequently shared links was labelled, which might have left out some alternative media sources that are much smaller in terms of readership in comparison with established media. The same goes for social media groups and accounts that also generate online content. However, we note that their prevalence is much more limited in comparison with other media resources and that social media pages and groups do not necessarily follow the same publishing logic as media resources. Another constraint of our analysis lies in the fact that it is difficult to disentangle different conversations that are part of the same thread, and identifying the timeframe for conversation entrainment can be performed in different ways. Our solution to account for this limitation was to test alternative time limits. Finally, there is also a possibility to test more complex statistical models beyond linear mixed-effects models to validate the results of our analysis.

Another limitation relates to resource labelling, which is in many ways a problematic task, since, as mentioned earlier, mainstream—alternative represents a continuum rather than a distinct set of categories, which makes it difficult to draw the boundary between different kinds of resources, and especially given that some alternative resources try to mimic the behaviour of their mainstream counterparts. Manual labelling also leaves some space for annotator subjectivity, especially with regard to the identification of oppositional or corrective stances. The latter is an especially difficult task, since it presumes deep contextual knowledge of the current public discourse on immigration. We try to mitigate this constraint by developing a multi-criterion labelling pipeline that enables a more consistent resource evaluation.

### 6.2 Implications

The existing research has also suggested that online platforms may enable users’ radicalization [77, 78] and provide discursive opportunities for right-wing violence [6], however, particular low-level mechanisms through which online platforms enable users’ radicalization remain under-investigated. In this study, we evaluated conversation dynamics, as well as users’ emotional and temporal alignment in particular, as possible factors that may pave the way for users’ more extreme views with regard to immigration policies. However, we have not found any evidence that conversations on Flashback´s subforum generate more negative or more coherent expressions about the topic. On the other hand, this observation partly speaks in favour of the earlier evidence that the emotional tone of online discussions can be best described as stable rather than fluctuating towards particular extremes [36], which highlights the need to explore alternative mechanisms through which the users become indoctrinated into more negative narratives.’

Our observations also call into question the effects of exposure to and consumption of alternative media content, and, in our empirical case, we notice no such effect with respect to the development of conversations, which also has some implications for the debate about the effect of alternative media content on its consumers and the effect of participation in the online fringe political groups. Specifically, we have focused on the distribution of different kinds of content, including mainstream or alternative media, as a particular conversation driver. We found that sharing any type of links has a limited effect only on users’ temporal alignment in the conversations, which is the only case when this effect is significant. Thus, turning back to the explanation offered by Collins’ IRC theory, link sharing can primarily be represented as an element of the collective symbol system [79], which is enabled by Flashback as an online platform, on par with, for example, specific jargon used to talk about immigrants (see e.g. [60]).

Further viewing our results through the prism of Collins’ theoretical approach, we have been able to detect what Collins describes in terms of participants’ entrainment, which, for him, is a necessary condition for a successful ritual [64]. We have evaluated two different ways to approximate such conversation entrainment, namely temporal alignment denoted by conversation pace and emotional alignment denoted by the dominating message sentiments. Based on our analysis, we find that temporal alignment seems to be a more straightforward way to uncover ritual entrainment. In other words, our interpretation is that, in online interaction rituals, synchronizing conversation rhymes is much easier than generating common moods, while the former nevertheless serves as an important condition for the ritual to occur. This observation is supported by Collins’ own remarks regarding the limits to which online interaction rituals can generate emotional energy [62].

One may explain the lack of emotional alignment simply by the fluctuation of user moods in the conversations. Although we know that the majority of users’ messages have, in general, negative appraisals of the immigration topic, this does not exclude the possibility that users’ emotions fluctuate in the level of negativity, or that users may abide by different opinions about what the best immigration policies should be. This may leave some space for users’ deliberations, a possibility that has been suggested by the earlier research [19]. Speaking of alternative media more specifically, one may also want to explore whether immigration-related content in such resources is more controversial, and thus generating more diverse emotional responses and expressions on the forum.

Finally, our results to some extent correspond to those of previous work that primarily focused on conversation length as a successful predictor of ritual’s success [50], which, once again, speaks in favour of users’ temporal alignment as one of the important indicators for the development of online interactions. On a more general level, we conclude that, in our empirical case, online communication has a limited possibility to enable users’ emotional mobilisation and spill over to offline contexts, which relates to the debate about the potential effects of right-wing platforms on users’ radicalisation.

## Supporting information

S1 File Labelling instructions. The document provides additional information on the instructions for resource labelling.
(PDF)

S2 File Resource labelling. The document provides additional information on the labelling of specific resources.
(XLSX)

## References

[1] Thorbjørnsrud K, Figenschou TU. The alarmed citizen: Fear, mistrust, and alternative media. Journalism Practice. 2022;16(5):1018–1035. doi: 10.1080/17512786.2020.1825113

[2] Östman K, Aschberg R. Flashback—ett laglöst land [Flashback—a lawless land]. Aftonbladet. 2015; 9 Feb. 2015. Available from: https://www.aftonbladet.se/nyheter/a/ddG3Rq/flashback--ett-laglost-land.

[3] Kaati L, Cohen K, Pelzer B, Akrami N, Andersson E, Knutas F. En studie i fördom: Om rasistiska stereotyper i digitala miljöer [A study in prejudice: On racial stereotypes in digital environments]. Totalförsvarets forskningsinstitut. 2022. Available from: https://www.foi.se/rest-api/report/FOI-R--5352--SE.

[4] Törnberg A, Törnberg P. Combining CDA and topic modeling: Analyzing discursive connections between Islamophobia and anti-feminism on an online forum. Discourse & Society. 2016;27(4):401–422. doi: 10.1177/0957926516634546

[5] Leung DKK, Lee FLF. Cultivating an active online counterpublic: Examining usage and political impact of Internet alternative media. The International Journal of Press/Politics. 2014;19(3):340–359. doi: 10.1177/1940161214530787

[6] Wahlström M, Törnberg A. Social media mechanisms for right-wing political violence in the 21st century: Discursive opportunities, group dynamics, and co-ordination. Terrorism and Political Violence. 2021;33(4):766–787. doi: 10.1080/09546553.2019.1586676

[7] Jiang B, Karami M, Cheng L, Black T, Liu H. Mechanisms and attributes of echo chambers in social media. arXiv preprint. 2021.

[8] Törnberg P. Echo chambers and viral misinformation: Modeling fake news as complex contagion. PLOS ONE. 2018;13(9):1–21. 10.1371/journal.pone.0203958 PMC6147442 30235239

[9] Theorin N, Strömbäck J. Some media matter more than others: Investigating media effects on attitudes toward and perceptions of immigration in Sweden. International Migration Review. 2020;54(4):1238–1264. doi: 10.1177/0197918319893292

[10] Collins R. Emotional energy as the common denominator of rational action. Rationality and Society. 1993;5(2):203–230. doi: 10.1177/1043463193005002005

[11] Schulze H. Who uses right-wing alternative online media? An exploration of audience characteristics. Politics and Governance. 2020;8(3):6–18. doi: 10.17645/pag.v8i3.2925

[12] Andersen K, Shehata A, Andersson D. Alternative news orientation and trust in mainstream media: A longitudinal audience perspective. Digital Journalism. 2021;0(0):1–20.

[13] Starbird K, Arif A, Wilson T, Van Koevering K, Yefimova K, Scarnecchia D. Ecosystem or echo-system? Exploring content sharing across alternative media domains. In: Proceedings of the International AAAI Conference on Web and Social Media. vol. 12; 2018.

[14] Sandberg LA, Ihlebæk KA. Start sharing the news. Statsvetenskaplig Tidskrift. 2019;121(3):421–440.

[15] Larsson AO. Right-wingers on the rise online: Insights from the 2018 Swedish elections. New Media & Society. 2020;22(12):2108–2127. doi: 10.1177/1461444819887700

[16] Hiaeshutter-Rice D, Weeks B. Understanding audience engagement with mainstream and alternative news posts on Facebook. Digital Journalism. 2021;9(5):519–548. doi: 10.1080/21670811.2021.1924068

[17] Haller A, Holt K. Paradoxical populism: How PEGIDA relates to mainstream and alternative media. Information, Communication & Society. 2019;22(12):1665–1680. doi: 10.1080/1369118X.2018.1449882

[18] Törnberg A, Nissen A. Mobilizing against Islam on social media: Hyperlink networking among European far-right extra-parliamentary Facebook groups. Information, Communication & Society. 2022; p. 1–19.

[19] Miro CJ, Toff B. How right-wing populists engage with cross-cutting news on online message boards: The case of ForoCoches and Vox in Spain. The International Journal of Press/Politics. 0;0(0):19401612211072696.

[20] Zannettou S, Caulfield T, De Cristofaro E, Kourtelris N, Leontiadis I, Sirivianos M, et al. The web centipede: Understanding how web communities influence each other through the lens of mainstream and alternative news sources. In: Proceedings of the 2017 Internet Measurement Conference; 2017. p. 405–417.

[21] Wang Y, Zannettou S, Blackburn J, Bradlyn B, De Cristofaro E, Stringhini G. A multi-platform analysis of political news discussion and sharing on web communities. In: 2021 IEEE International Conference on Big Data (Big Data); 2021. p. 1481–1492.

[22] Luna JP, Toro S, Valenzuela S. Amplifying counter-public spheres on social media: News sharing of alternative versus traditional media after the 2019 Chilean uprising. Social Media + Society. 2022;8(1):20563051221077308. doi: 10.1177/20563051221077308

[23] Introne J, Iandoli L, DeCook J, Yildirim IG, Elzeini S. The collaborative construction and evolution of pseudo-knowledge in online conversations. In: Proceedings of the 8th International Conference on Social Media & Society. New York, NY, USA: Association for Computing Machinery; 2017. Available from: 10.1145/3097286.3097297.

[24] Cai M, Luo H, Meng X, Cui Y, Wang W. Influence of information attributes on information dissemination in public health emergencies. Humanities and Social Sciences Communications. 2022;9(1):1–22. doi: 10.1057/s41599-022-01278-2 35967483 PMC9361962

[25] Wei J, Zhang L. Analysis of information dissemination based on emotional and the evolution life cycle of public opinion. In: 2019 International Conference on Robots Intelligent System (ICRIS); 2019. p. 265–268.

[26] Maros A, Almeida JM, Vasconcelos M. A Study of misinformation in audio messages shared in WhatsApp groups. In: Bright J, Giachanou A, Spaiser V, Spezzano F, George A, Pavliuc A, editors. Disinformation in Open Online Media. Cham: Springer International Publishing; 2021. p. 85–100.

[27] Paschen J. Investigating the emotional appeal of fake news using artificial intelligence and human contributions. Journal of Product & Brand Management. 2020;29(2):223–233. doi: 10.1108/JPBM-12-2018-2179

[28] Tuomola S, Wahl-Jorgensen K. Emotion mobilisation through the imagery of people in Finnish-language right-wing alternative media. Digital Journalism. 2023;11(1):61–79. doi: 10.1080/21670811.2022.2061551

[29] Gerbaudo P, Falco CCD, Giorgi G, Keeling S, Murolo A, Nunziata F. Angry posts mobilize: Emotional communication and online mobilization in the Facebook pages of Western European right-wing populist leaders. Social Media + Society. 2023;9(1):20563051231163327. doi: 10.1177/20563051231163327

[30] Berger J, Milkman KL. What makes online content viral? Journal of Marketing Research. 2012;49(2):192–205. doi: 10.1509/jmr.10.0353

[31] Garcia D, Kappas A, Küster D, Schweitzer F. The dynamics of emotions in online interaction. Royal Society Open Science. 2016;3(8):160059. doi: 10.1098/rsos.160059 27853586 PMC5108936

[32] Kramer ADI, Guillory JE, Hancock JT. Experimental evidence of massive-scale emotional contagion through social networks. Proceedings of the National Academy of Sciences. 2014;111(24):8788–8790. doi: 10.1073/pnas.1320040111 24889601 PMC4066473

[33] Ferrara E, Yang Z. Measuring emotional contagion in social media. PLOS ONE. 2015;10(11):e0142390. doi: 10.1371/journal.pone.0142390 26544688 PMC4636231

[34] Fan R, Zhao J, Chen Y, Xu K. Anger is more influential than joy: Sentiment correlation in Weibo. PLOS ONE. 2014;9(10):e110184. doi: 10.1371/journal.pone.0110184 25333778 PMC4198202

[35] Fan R, Xu K, Zhao J. Higher contagion and weaker ties mean anger spreads faster than joy in social media. arXiv preprint arXiv:160803656. 2016;.

[36] Garas A, Garcia D, Skowron M, Schweitzer F. Emotional persistence in online chatting communities. Scientific Reports. 2012;2(1):1–8. doi: 10.1038/srep00402 22577512 PMC3349267

[37] Katharina Esau JB Lena Wilms, Keller B. For Deliberation Sake, Show Some Constructive Emotion! How Different Types of Emotions Affect the Deliberative Quality of Interactive User Comments. Javnost—The Public. 2023;0(0):1–24.

[38] Song Y, Dai XY, Wang J. Not all emotions are created equal: Expressive behavior of the networked public on China’s social media site. Computers in Human Behavior. 2016;60:525–533. doi: 10.1016/j.chb.2016.02.086

[39] Momennejad I, Duker A, Coman A. Bridge ties bind collective memories. Nature Communications. 2019;10(1):1–8. doi: 10.1038/s41467-019-09452-y 30952861 PMC6451000

[40] Leal H. Networked disinformation and the lifecycle of online conspiracy theories. In: Butter M, Knight P, editors. Routledge Handbook of Conspiracy Theories. Routledge; 2020. p. 497–511.

[41] De Choudhury M, Sundaram H, John A, Seligmann DD. What makes conversations interesting? Themes, participants and consequences of conversations in online social media. In: Proceedings of the 18th International Conference on World Wide Web; 2009. p. 331–340.

[42] Backstrom L, Kleinberg J, Lee L, Danescu-Niculescu-Mizil C. Characterizing and curating conversation threads: Expansion, focus, volume, re-entry. In: Proceedings of the Sixth ACM International Conference on Web Search and Data Mining; 2013. p. 13–22.

[43] Bao J, Wu J, Zhang Y, Chandrasekharan E, Jurgens D. Conversations gone alright: Quantifying and predicting prosocial outcomes in online conversations. In: Proceedings of the Web Conference 2021; 2021. p. 1134–1145.

[44] de Kock C, Vlachos A. I Beg to Differ: A study of constructive disagreement in online conversations. ArXiv preprint. 2021.

[45] Aragón P, Gómez V, Kaltenbrunner A. To thread or not to thread: The impact of conversation threading on online discussion. In: Eleventh International AAAI Conference on Web and Social Media. vol. 11;. p. 12–21.

[46] Bagavathi A, Bashiri P, Reid S, Phillips M, Krishnan S. Examining untempered social media: Analyzing cascades of polarized conversations. In: Proceedings of the 2019 IEEE/ACM International Conference on Advances in Social Networks Analysis and Mining. ASONAM’19. New York, NY, USA: Association for Computing Machinery; 2020. p. 625–632. Available from: 10.1145/3341161.3343695.

[47] Wang C, Ye M, Huberman BA. From user comments to on-line conversations. In: Proceedings of the 18th ACM SIGKDD international conference on Knowledge discovery and data mining; 2012. p. 244–252.

[48] Chen J, Wang C, Lin H, Wang W, Cai Z, Wang J. Learning the structures of online asynchronous conversations. In: Database Systems for Advanced Applications: 22nd International Conference, DASFAA 2017, Suzhou, China, March 27-30, 2017, Proceedings, Part I 22. Springer; 2017. p. 19–34.

[49] Caetano JA, Magno G, Gonçalves M, Almeida J, Marques-Neto HT, Almeida V. Characterizing attention cascades in WhatsApp groups. In: Proceedings of the 10th ACM Conference on Web Science. ACM; 2019. Available from: https://doi.org/10.1145%2F3292522.3326018.

[50] DiMaggio P, Bernier C, Heckscher C, Mimno D. Interaction ritual threads: Does IRC theory apply online? In: Ritual, Emotion, Violence. Routledge; 2018. p. 81–124.

[51] Van Haperen S, Uitermark J, Van der Zeeuw A. Mediated interaction rituals: A geography of everyday life and contention in Black Lives Matter. Mobilization: An International Quarterly. 2020;25(3):295–313. doi: 10.17813/1086-671X-25-3-295

[52] Von Scheve C, Salmella M. Collective emotions: Perspectives from Psychology, Philosophy, and Sociology. OUP Oxford; 2014.

[53] Collins R. On the microfoundations of macrosociology. American Journal of Sociology. 1981;86(5):984–1014. doi: 10.1086/227351

[54] Sacks H, Schegloff EA, Jefferson G. A simplest systematics for the organization of turn taking for conversation. In: Studies in the Organization of Conversational Interaction. Elsevier; 1978. p. 7–55.

[55] Goodwin C, Heritage J. Conversation analysis. Annual Review of Anthropology. 1990;19(1):283–307. doi: 10.1146/annurev.an.19.100190.001435

[56] Kemper TD. Status, Power and Ritual Interaction: A Relational Reading of Durkheim, Goffman and Collins. Routledge; 2016.

[57] Collins R. Interaction Ritual Chains. Princeton Studies in Cultural Sociology; 62. Princeton, NJ: Princeton University Press; 2014.

[58] Habermas J. Political communication in media society: Does democracy still enjoy an epistemic dimension? The impact of normative theory on empirical research. Communication Theory. 2006;16(4):411–426. doi: 10.1111/j.1468-2885.2006.00280.x

[59] Van Dijk TA. Stories and racism. Narrative and Social Sontrol: Critical Perspectives. 1993;21:121–142.

[60] Åkerlund M. Dog whistling far-right code words: The case of ‘culture enricher’ on the Swedish web. Information, Communication & Society. 2022;25(12):1808–1825. doi: 10.1080/1369118X.2021.1889639

[61] Collins R. Interaction ritual chains and collective effervescence. In: von Scheve C, Salmella M, editors. Collective Emotions. Oxford University Press; 2014. p. 299–311.

[62] Collins R. Social distancing as a critical test of the micro-sociology of solidarity. American Journal of Cultural Sociology. 2020;8(3):477–497. doi: 10.1057/s41290-020-00120-z 33101676 PMC7575701

[63] Collins R, Hanneman R. Modelling the interaction ritual theory of solidarity. In: Fararo TJ, Doreian P, editors. The Problem of Solidarity: Theories and Models. Amsterdam: Gordon and Breach Publishers; 1998. p. 213–237.

[64] Collins R. The mutual-focus / emotional-entrainment model. In: Interaction Ritual Chains. Princeton University Press; 2004. p. 47–101.

[65] Wickham H. httr: Tools for Working with URLs and HTTP; 2023.

[66] Vega D, Magnani M. In: Holme P, Saramäki J, editors. Metrics for Temporal Text Networks. Computational Social Sciences. Springer; 2019. p. 147–160.

[67] Hagberg A, Swart P, S Chult D. Exploring network structure, dynamics, and function using NetworkX. Los Alamos National Lab.(LANL), Los Alamos, NM (United States); 2008.

[68] Du Bois J. The stance triangle. In: Englebretson R, editor. Stancetaking in Discourse: Subjectivity, Evaluation, Interaction; 2007. p. 139–182.

[69] Küçük D, Can F. Stance detection: A survey. ACM Comput Surv. 2020;53(1).

[70] Yantseva V, Kucher K. Stance classification of social media texts for under-resourced scenarios in social sciences. Data. 2022;7(11):159. doi: 10.3390/data7110159

[71] Hutto CJ, Gilbert E. VADER: A parsimonious rule-based model for sentiment analysis of social media text. Proceedings of the 8th International Conference on Weblogs and Social Media, ICWSM 2014. 2015; p. 216–225.

[72] Kopacheva E, Yantseva V. Users’ polarisation in dynamic discussion networks: The case of refugee crisis in Sweden. PLOS ONE. 2022;17(2):1–30. doi: 10.1371/journal.pone.0262992 35139109 PMC8827437

[73] Holt K, Figenschou TU, Frischlich L. Key dimensions of alternative news media. Digital Journalism. 2019;7(7):860–869. doi: 10.1080/21670811.2019.1625715

[74] Bates D, Mächler M, Bolker B, Walker S. Fitting linear mixed-effects models using lme4. Journal of Statistical Software. 2015;67(1):1–48. doi: 10.18637/jss.v067.i01

[75] Hamaker EL, Grasman RPPP. To center or not to center? Investigating inertia with a multilevel autoregressive model. Frontiers in Psychology. 2015;5. doi: 10.3389/fpsyg.2014.01492 PMC4310502 25688215

[76] Knobloch-Westerwick S, Mothes C, Polavin N. Confirmation bias, ingroup bias, and negativity bias in selective exposure to political information. Communication Research. 2020;47(1):104–124. doi: 10.1177/0093650217719596

[77] Riebe T, Pätsch K, Kaufhold MA, Reuter C. From conspiracies to insults: A case study of radicalisation in social media discourse. In: Dachselt R, Weber G, editors. Mensch und Computer 2018—Workshopband. Bonn: Gesellschaft für Informatik e.V.; 2018.

[78] Odag Ö, Leiser A, Boehnke K. Reviewing the role of the Internet in radicalization processes. Journal for deradicalization. 2019;(21):261–300.

[79] Rössel J, Collins R. Conflict theory and interaction rituals: The microfoundations of conflict theory. In: Turner JH, editor. Handbook of Sociological Theory. Springer; 2001. p. 509–531.

