# Supplementary material for: Immigrant-critical alternative media in online conversations
Source: PLoS One. 2023 Nov 30;18(11):e0294636. doi: 10.1371/journal.pone.0294636 (PMC10688883; doi:10.1371/journal.pone.0294636)
Supplement: S1 File — The document provides additional information on the instructions for resource labelling. (PDF) [file pone.0294636.s001.pdf]

Appendix 1: Codebook for media resource labelling

Source selection

As a first step, we extract all relative URLS from the text messages and clean them in such a way that only base URLS (e.g. www.example.com) are left, and then count the number of times each clean URL occurrs in the dataset. For the subsequent source classification, we select most quoted resources that account for 75% of all URLs shared on the subforum. Further, we filter out all "non-media" resources (websites of private companies, non-profit and governmental organisations, as well as web services such as e.g. photo hosting websites). After filtering, we obtain a list of 95 media resources.

Source type identification

The resulting resources are labelled as belonging to one of the three categories: *mainstream (legacy)* (or in short MM), *immigrant-critical alternative* (hereafter AM) and *other*, since, as pointed by the earlier research, the distinction between alternative and mainstream media can be best described in terms of a continuum rather than distinctly existing categories (Holt et al. 2019). We note, however, that not all resources in our media list can be described as "news media". Thus, instead of "news media" we use the notion of "media resources" to denote the variability of the media content and formats.

For the task of classifying particular source types, we follow Holt et al. (2019) who identifies several dimensions of "alternativeness", in particular, with regard to the *content*, *producers* and *organizations* (ibid.).

According to Holt et al.: "Alternative news media represent a proclaimed and/or (self-) perceived corrective, opposing the overall tendency of public discourse emanating from what is perceived as the dominant mainstream media in a given system" (ibid.). Thus, alternative media content disseminates narratives that are perceived as counter-hegemonic and marginalized in the mainstream discourse. Alternative news producers include, in particular, non- or semi-professional actors, such as readers and activists; and, finally, alternative news organisations comprise non-conventional communication formats and strategies, in particular, in low-cost formats, such as blogs and webpages (ibid.).

Based on these dimensions, we identify several binary criteria that each resource is checked against. We manually access each resource's URL and check it's scoring on the criteria described below (YES if the criterion is fulfilled, and NO otherwise; NA is used when information is unavailable). If necessary, we search information about the resource on the Web if it is not directly available on the resource's website (e.g. as in cases resource ownership or adherence to press ethical standards, see below).

Non-oppositional stance with regard to the immigration topic, as described by Holt et al. (2019). The latter is identified either through the qualitative reading and assessment of the actual media content published on the resource or through studying the resource's self-ascribed role (e.g. through reading "About us" pages).

Availability in conventional (high-cost) format. High-cost formats include printed press, television or national broadcasting radio formats.

Adherence to press and journalist ethics standards. For the Nordic resources, we check whether the resource is a member of the national press-ethical system:

Sweden: Sveriges Tidskrifter (https://sverigestidskrifter.se/vara-medlemmar/), Tidningsutgivarna (https://tu.se/) or Medieombudsmannen (https://medieombudsmannen.se/hela-listan-over-frivilligt-anslutna-medier/)

Norway: Medietilsynet (https://www.medietilsynet.no/)

Denmark: Pressenævnet (https://www.pressenaevnet.dk/hvem-kan-man-klage-over/)

Finland: JUSANEK (https://jsn.fi/sv/vad-ar-onm/jusanek-se/jusaneks-medlemmar/) or Uutismedian liitto (https://www.uutismediat.fi/sanomalehtihaku/)

For the rest of the world, we check whether resources have ethical statements on their websites.

The existence of an Editor-in-chief and/or Editorial board. We check whether the information about employees responsible for the published content (the Editor-in-chief and/or Editorial board) is openly available on the website.

Affiliation with a larger publisher or organization. We check whether a resource is part of or owned by a larger organization, such as publishing house, or larger business in general, or is publicly owned or owned by the government.

Other coding instructions

- In cases when a resource is not available, we use Wayback Machine (https://web.archive.org/) to check the resource content.
- For the paid content or the content that requires subscription, we use, where possible, Retriever MedieArkivet (https://www.retrievergroup.com/sv/product-mediearkivet) provided by Uppsala University to check such content. Otherwise such content is omitted from the evaluation.
- For the resources in languages other than Swedish or English, we use Google Translate function inbuilt in the web-browser.

References

Kristoffer Holt, Tine Ustad Figenschou & Lena Frischlich (2019) Key Dimensions of Alternative News Media, Digital Journalism, 7:7, 860-869, DOI: 10.1080/21670811.2019.1625715.
